# Determinants of social inclusion and their effect on the wellbeing of older adults

**DOI:** 10.1093/geroni/igaa057.1021

**Published:** 2020-12-16

**Authors:** Keya Sen, Victor Prybutok, Gayle Prybutok

**Affiliations:** 1 University of North Texas, Denton, Texas, United States; 2 UNT, Denton, Texas, United States; 3 UNT, Denton, United States

## Abstract

Social inclusion fosters interpersonal relationships that reduce social isolation and enhance wellbeing in older adults. This study finds that socially engaged older adults are less likely to decline in health and have more wellbeing than those less engaged. The connection between wellbeing and social engagement is examined with hypotheses that there is a significant linear relationship between wellbeing and age, ethnicity, gender, the involvement and perception of participatory activities, community dwelling and the use of technology among older adults. A multiple linear regression on 4621samples obtained from National Health and Aging Trend Study, Round 8 shows that social engagement explained a unique variance in wellbeing (34.5%) suggesting that more social connections, via social activities, community-dwelling, mobility, and use of technology, there is enhanced health and fewer chances of cognitive decline in older adults. The use of text messaging and emails had a moderating effect on cognition and wellbeing of older adults. It is suggested that existing low-cost community programs targeting the so-called social determinants of health can be reworked to address social isolation and foster knowledge and technology skills in the older population. Directions for future research include examining human behaviors and perceptions to stay connected.

